# Supplementary material for: A qualitative evaluation of a global surgery course within the University of Cape Town’s master of public health curriculum: A cross-sectional study
Source: PLOS Glob Public Health. 2025 Dec 12;5(12):e0005646. doi: 10.1371/journal.pgph.0005646 (PMC12700414; doi:10.1371/journal.pgph.0005646)
Supplement: S1 Appendix — (DOCX) [file pgph.0005646.s001.docx]

**S1 Appendix: Interview Guide**

*Section 1: Participant Demographics*

1. Year of participation:
2. What is your age group? (Select one)

- 20–29 years
- 30–39 years
- 40–49 years
- 50–59 years
- 60+ years

1. What is your gender? (Select one)

- Male
- Female
- Non-binary/Other
- Prefer not to say

1. Describe your professional background
2. What is your country of origin? (Drop-down list of countries)
3. What is your current workplace setting?
4. How many years of experience do you have in your field? (Select one)

- 0–5 years
- 6–10 years
- 11–15 years
- 16+ years

*Section 2.1: Background & Motivation*

1. Can you tell me a bit more about your professional background and what led you to enrol in the MPH Global Surgery course?
2. What were your expectations when you joined the course?
3. How did you first hear about the program, and what factors influenced your decision to participate?

*Section 2.2: Learning Experience & Curriculum*

1. Which components of the Global Surgery course curriculum did you find most useful or memorable? Why?
2. How did the content relate to your current or previous professional responsibilities?
3. Were there any aspects of the Global Surgery course you found particularly challenging? How did you navigate them?
4. Can you describe any moments during the course where your perspective on surgical systems and/or public health shifted?

*Section 2.3: Application & Professional Impact*

1. Since completing the Global Surgery course, how have you applied the Global Surgery knowledge or skills gained in your professional setting?
2. Has your role changed in any way since finishing the course—such as taking on new responsibilities, leadership roles, or projects?
3. How do you incorporate the principles and frameworks of global surgery you learned as well as the insights on surgical systems into your daily work or strategic decisions?

*Section 2.4: Program Delivery & Suggestions for Improvement*

1. How would you describe the learning format and delivery of the course? What worked well, and what could be improved?
2. Were there any barriers or difficulties you faced while completing the course (e.g., access to materials, balancing work and study)?
3. What additional topics, formats, or support do you think would strengthen the course’s impact?

*Section 2.5: Broader Impact & Reflections*

1. Has this program influenced how you view the role of surgery in public health or health systems strengthening? If so, how?
2. Would you recommend the course to colleagues in your field? Why or why not?
3. Is there anything else you’d like to share about your experience in the course or how it has shaped your career or thinking?
