# Supplementary material for: A qualitative evaluation of a global surgery course within the University of Cape Town’s master of public health curriculum: A cross-sectional study
Source: PLOS Glob Public Health. 2025 Dec 12;5(12):e0005646. doi: 10.1371/journal.pgph.0005646 (PMC12700414; doi:10.1371/journal.pgph.0005646)
Supplement: S2 Appendix — (DOCX) [file pgph.0005646.s002.docx]

**S2 Appendix: COREQ Checklist: A 32-Item Checklist for Interviews and Focus Groups**

| No. | Item | Guide Questions/Description | Reported on Page / Line No. (or in Manuscript Section) |
| --- | --- | --- | --- |
| 1 | Interviewer/facilitator | Which author(s) conducted the interview or focus group? | Methods: Data Collection and Management - The main author (Yvan Zolo) conducted all interviews. |
| 2 | Credentials | What were the researcher's credentials? (e.g., PhD, MD) | Authors' Affiliations - All authors hold advanced degrees and are affiliated with academic or professional institutions. |
| 3 | Occupation | What was their occupation at the time of the study? | Authors' Affiliations; Methods: Research Team - The interviewer was a researcher within the Global Surgery Division. |
| 4 | Gender | Was the researcher male or female? | Methods: Research Team, Reflexivity - The interviewer is male. |
| 5 | Experience and training | What experience or training did the researcher have? | Methods: Research Team, Reflexivity - The research team included experts in Global Surgery, Public Health, and medical education. |
| 6 | Relationship established | Was a relationship established prior to study commencement? | Methods: Research Team, Reflexivity - The interviewer was not involved in the design or instruction of the course, so no prior relationship with participants as their instructor existed. |
| 7 | Participant knowledge of the interviewer | What did the participants know about the researcher? (e.g., personal goals, reasons for doing the research) | The information sheet and consent form provided to participants detailed the purpose of the research and the researcher's role. |
| 8 | Interviewer characteristics | What characteristics were reported about the interviewer/facilitator? (e.g., Bias, assumptions, reasons and interests in the research topic) | Methods: Research Team, Reflexivity - It is stated that the interviewer was not involved in the course design or instruction to mitigate bias. The team's expertise is reported. |
| 9 | Methodological orientation and Theory | What methodological orientation was stated to underpin the study? | Methods: Research Team, Reflexivity and Data Analysis - The study used a qualitative descriptive design and thematic analysis following Braun and Clarke. |
| 10 | Sampling | How were participants selected? | Methods: Participant Selection and Recruitment - A purposive sample was recruited. |
| 11 | Method of approach | How were participants approached? | Methods: Participant Selection and Recruitment - Participants were contacted by email and phone. |
| 12 | Sample size | How many participants were in the study? | Results - Ten alumni participated. |
| 13 | Non-participation | How many people refused to participate or dropped out? Reasons? | Not explicitly stated, but the recruitment target (8-12 from a pool of 20) was met. |
| 14 | Setting of data collection | Where was the data collected? | Methods: Data Collection and Management - Interviews were conducted online via Zoom, with both interviewer and participants in private settings. |
| 15 | Presence of non-participants | Was anyone else present besides the participants and researchers? | Methods: Data Collection and Management - Participants were instructed to find a private location to ensure confidentiality, implying no one else was present. |
| 16 | Description of sample | What are the important characteristics of the sample? | Results; Table 1 - Participant demographics, professional roles, experience, and country of origin are provided in detail. |
| 17 | Interview guide | Were questions, prompts, guidelines provided by the authors? Was it pilot tested? | Methods: Data Collection and Management; Appendix - The interview guide was developed, reviewed by experts, pilot-tested, and is provided in the appendix. |
| 18 | Repeat interviews | Were repeat interviews carried out? If yes, how many? | No repeat interviews were conducted. |
| 19 | Audio/visual recording | Did the research use audio or visual recording to collect the data? | Methods: Data Collection and Management - Sessions were audio-recorded with consent. |
| 20 | Field notes | Were field notes made during and/or after the interview or focus group? | Not explicitly stated. |
| 21 | Duration | What was the duration of the interviews or focus group? | Methods: Data Collection and Management - Sessions lasted 35-40 minutes each. |
| 22 | Data saturation | Was data saturation discussed? | Methods: Participant Selection and Recruitment - Sampling continued until thematic saturation was achieved. |
| 23 | Transcripts returned | Were transcripts returned to participants for comment and/or correction? | Not mentioned in the manuscript because they were not. |
| 24 | Number of data coders | How many data coders coded the data? | Methods: Research Team, Reflexivity and Data Analysis - The main author (Yvan Zolo) conducted the analysis. Co-author Moses Isiagi is listed under 'Data curation, formal analysis.' |
| 25 | Description of the coding tree | Did authors provide a description of the coding tree? | Results - The five central themes and sub-themes are described in detail, effectively presenting the coding structure. |
| 26 | Derivation of themes | Were themes identified in advance or derived from the data? | Methods: Research Team, Reflexivity and Data Analysis - Thematic analysis was conducted to identify emergent themes. |
| 27 | Software | What software, if applicable, was used to manage the data? | Methods: Research Team, Reflexivity and Data Analysis - NVivo software was used. |
| 28 | Participant checking | Did participants provide feedback on the findings? | Not mentioned in the manuscript because we did not seek their feedback. |
| 29 | Quotations presented | Were participant quotations presented to illustrate the themes/findings? Was each quotation identified? | Results - Numerous direct quotations from participants (identified as P1, P2, etc.) are provided throughout the thematic results. |
| 30 | Data and findings consistent | Was there consistency between the data presented and the findings? | Yes, the findings (themes) are directly supported by the quoted data in the results section. |
| 31 | Clarity of major themes | Were major themes clearly presented in the findings? | Results - Five central themes are clearly defined and described with sub-themes. |
| 32 | Clarity of minor themes | Is there a description of diverse cases or discussion of minor themes? | Results - Sub-themes capture nuances and variations, and Table 2 provides detailed evidence of application, showing diversity of impact. |
