# Supplementary material for: A qualitative evaluation of a global surgery course within the University of Cape Town’s master of public health curriculum: A cross-sectional study
Source: PLOS Glob Public Health. 2025 Dec 12;5(12):e0005646. doi: 10.1371/journal.pgph.0005646 (PMC12700414; doi:10.1371/journal.pgph.0005646)
Supplement: S1 Transcripts — (DOCX) [file pgph.0005646.s003.docx]

**S1 Transcripts: Anonymized Participant Interview Transcripts**

This document contains the cleaned and anonymized interview transcripts for the ten participants in the study. Transcripts are structured by participant and organized according to the main themes of the interview guide. Participant identifiers (P1-P10) correspond to those presented in Table 1 of the main manuscript.

**Participant P1 Interview Transcript**

Participant Demographics:

- Age group: 30–39

- Gender: Male

- Professional background: Medical doctor, clinical practice

- Country of origin: Malawi

- Current workplace setting: University

- Years of experience: 6–10 years

- Year of Course Completion: 2024

Section 2.1 – Background and Motivation

Professional background and reason for enrolling in the course:

I'm a medical doctor by profession from Malawi. I enrolled in the Global Surgery course mainly out of curiosity, because I had no prior knowledge of global surgery. I wanted to understand what it entailed and how it intersects with public health.

Expectations before joining the course:

I expected to get a good understanding of what global surgery is all about, since it was a completely new concept to me.

How you first heard about the course and what influenced your decision to join:

I first heard about the course from a student in the MSc in Global Surgery program. He told me the course was also available for MPH students. Later, my supervisor also recommended it. Both of these influenced me—along with my curiosity—to explore what global surgery was.

Section 2.2 – Learning Experience and Curriculum

Most useful or memorable curriculum components:

I really enjoyed the case-based discussions. For example, we’d be given a scenario like an accident in an underserved community and asked to discuss how to approach it. These practical, interactive sessions were the most engaging and helpful for me.

Relevance to previous or current responsibilities:

The content was very relatable. In my previous clinical work in Malawi, I was involved in surgery. This course helped me reflect more holistically on patients—not just the condition, but also where they come from and their broader context.

Challenging aspects and how you managed them:

The only real challenge was the exam—there was a lot of writing, which I found a bit overwhelming.

Moments of shift in perspective:

Yes—especially during a topic on green surgery. It made me realize that expanding surgical access also has environmental implications, which I hadn’t considered before. It shifted my thinking beyond just access to include sustainability.

Section 2.3 – Application and Professional Impact

How you have applied the knowledge/skills gained:

Since the course, I’ve transitioned into public health and global surgery research. I use a lot of what I learned—especially the frameworks—in my current role. In fact, I joined the Global Surgery Department, where I apply these skills daily.

Changes in your role since completing the course:

Yes. I’m now a research fellow in the Global Surgery Department, working on major projects and taking on more responsibilities than before.

Examples of current projects:

1. The Stillbirth Africa project, in partnership with multiple international organizations. We’re advocating for attention to stillbirths, which are a highly neglected condition across Africa with poor reporting systems.

2. A project with a health economics department focusing on critical and essential retrospective surgical care in Africa.

Incorporating surgical systems knowledge in daily work or decisions:

Absolutely. My thinking about surgery is now broader. I consider not only the clinical aspect but also social determinants and public health strategies. I use frameworks from the course in project management and system-level thinking.

Section 2.4 – Program Delivery and Suggestions

How you found the learning format and delivery:

The case-based discussions worked very well. They were interactive, with faculty joining us. We were also assigned two projects—one was a perspective piece, which I found especially valuable.

One downside: some classes were scheduled late in the day, which was challenging when we were already mentally tired.

Any barriers or difficulties faced during the course:

No, I didn’t face any major difficulties—access to materials and balancing work was manageable.

Suggestions for strengthening the course:

As a health economist, I felt there wasn’t enough coverage of the economic burden of surgery—on patients and health systems. This could be better addressed in future iterations.

Section 2.5 – Broader Impact and Reflection

Has your view of the role of surgery in public health changed?

Yes, completely. I now advocate for global surgery. It’s not just about the “knife”—it’s about system-wide thinking and using a public health lens to make surgical care more impactful.

Would you recommend the course to colleagues?

Yes, and I’ve already done so. It changed my perspective and was one of the best modules I took during my MPH.

Any final reflections:

I’m grateful for the experience. It opened doors for me and was probably my favorite module. It helped shape my career trajectory.

**Participant P2 Interview Transcript**

Participant Demographics:

- Age group: 20–29

- Gender: Female

- Professional background: Academic research

- Country of origin: South Africa

- Current workplace setting: University / Private research society

- Years of experience: 0-5 years in research (6-10 in healthcare overall)

- Year of Course Completion: 2024

Section 2.1 – Background and Motivation

Professional background and reason for enrolling in the course:

My professional background is in health sciences and biokinetics. I developed a huge passion for maternal and child health and enrolled in a master's in Epidemiology and Biostatistics. I enrolled in the course very spontaneously. I didn’t know global surgery was a discipline. It was introduced during orientation week. The description in the booklet interested me. It was curiosity that drove me to apply.

Expectations before joining the course:

I was very curious and wanted to learn. I was interested to see how someone without a surgical background would fit into a global surgery class. I knew I’d feel like an imposter, but I was up for the challenge. My past work with moms and babies also motivated me, especially seeing gaps in surgical care for them.

How you first heard about the course and what influenced your decision to join:

I heard about it during orientation week.

Section 2.2 – Learning Experience and Curriculum

Most useful or memorable curriculum components:

Leadership classes really stuck with me and changed my perspective. Implementation science was new for me and also very valuable. Advocacy was another key component—I appreciated learning about advocating for surgical care in public health. The course unpacked how surgery fits into public health, which I found fascinating.

Relevance to previous or current responsibilities:

Leadership and advocacy are more present in my work now. I’ve had field experience in data collection and analysis. Now, I encourage patients to advocate for themselves, and I support them in that.

Challenging aspects and how you managed them:

Implementation science and a few clinical concepts were new to me. I had to explore and learn more on my own. Also, balancing in-person classes with work was difficult. I’d suggest a hybrid option for future delivery.

Moments of shift in perspective:

Yes, by the end of the course, especially during the final assignment, I realized how surgical systems fit into public health. The course built on this understanding over time.

Section 2.3 – Application and Professional Impact

How you have applied the knowledge/skills gained:

I’ve taken on more leadership and advocacy roles in my research setting. I feel more confident to lead and advocate.

Changes in your role since completing the course:

Yes, I’ve taken on more roles in evidence-based research like scoping reviews. I’m collecting data for two projects. One is on healthcare workers’ knowledge and perceptions of vaccines. The second is a neuroscience project studying child development and socioeconomic factors.

Incorporating surgical systems knowledge in daily work or decisions:

Not directly in data collection, but in evidence-based research, I assess whether studies are equitable, scalable, and sustainable—principles emphasized in the global surgery course.

Section 2.4 – Program Delivery and Suggestions

How you found the learning format and delivery:

The format worked well. Slides were helpful, but I really liked the interactive discussions and how conveners tied in personal and student experiences. Assignments like presentations, opinion pieces, and protocols were valuable. I’d suggest offering a hybrid format for flexibility.

Any barriers or difficulties faced during the course:

Balancing work and studies was tough. Adjusting to a new city and university was also a challenge. But materials were always accessible.

Suggestions for strengthening the course:

I’d suggest including a bit of epidemiology—especially for those not taking the epidemiology course.

Section 2.5 – Broader Impact and Reflection

Has your view of the role of surgery in public health changed?

Yes, it changed my view of surgery from being a purely clinical service to a critical public health component. My perspective now includes surgery alongside vaccines and other public health interventions.

Would you recommend the course to colleagues?

Absolutely. I’ve already recommended it to others—both clinical and non-clinical. It’s valuable for everyone. I genuinely learned a lot and want others to benefit too.

Any final reflections:

I’ve probably spoken too much already! But if anything is unclear in the transcript, please reach out—I’m happy to clarify.

**Participant P3 Interview Transcript**

Participant Demographics:

- Age group: 30–39

- Gender: Male

- Professional background: Academic research, public health, biomedical science

- Country of origin: South Africa

- Current workplace setting: Urban hospital

- Years of experience: 6-10 years

- Year of Course Completion: 2023

Section 2.1 – Background and Motivation

Professional background and reason for enrolling in the course:

My background is biomedical science, public health, and academic research. I’m not a clinician. I first heard about the course from a colleague at UCT who mentioned there was an MSc in Global Surgery and an course module. Before that, I had already been involved in multi-country research, which made me think I needed to do something related to global surgery.

Expectations before joining the course:

I expected to gain more practical knowledge on global surgery challenges. I remembered the Lancet Global Surgery paper and wanted to learn more about improving surgical access in low-income countries, build my data analysis skills, understand health systems, and prepare myself for leadership roles.

How you first heard about the course and what influenced your decision to join:

I heard about it from a colleague at UCT. My prior involvement in multi-country research was a key influencing factor.

Section 2.2 – Learning Experience and Curriculum

Most useful or memorable curriculum components:

Surgical Systems Strengthening and Health Policy were the most useful. They provided real-world case studies from Africa, directly applicable to my context. I also remember a class discussion on challenges in theatres — those practical exchanges helped me learn a lot.

Relevance to previous or current responsibilities:

It helped me think about data-driven decision-making and tracking surgical outcomes in rural areas. I applied lessons to refine processes in my work and learned the importance of advocating for policy changes to improve health systems.

Challenging aspects and how you managed them:

Balancing full-time work with the course was tough — readings, assignments, and classes were demanding. I managed by setting aside evening study time and reaching out to the facilitator for extensions or extra materials. She was very supportive.

Moments of shift in perspective:

One moment that shifted my view was learning about the economic burden of untreated surgical conditions. It made me realize how interconnected surgery is with many other components of health systems.

Section 2.3 – Application and Professional Impact

How you have applied the knowledge/skills gained:

I’m now leading a project to map surgical access gaps using data analysis tools. We’re working on “failure to rescue” research. The course helped me grow from junior staff to someone applying system thinking to improve efficiency and resource allocation.

Changes in your role since completing the course:

I recently applied for a leadership position as Senior Chief Scientist. If approved, I’ll oversee projects and collaborate closely with health systems teams. The course gave me confidence and the tools to contribute at a higher level.

Incorporating surgical systems knowledge in daily work or decisions:

I use frameworks from the course to evaluate resources and to improve teamwork among surgeons, anaesthetists, and nurses. Having learned about conflict resolution in class helps me navigate theatre dynamics.

Section 2.4 – Program Delivery and Suggestions

How you found the learning format and delivery:

I appreciated having slides and videos uploaded before class. Watching pre-recorded lectures beforehand reduced my workload and improved my participation.

Any barriers or difficulties faced during the course:

Balancing full-time work with the course was the main challenge.

Suggestions for strengthening the course:

Add mentorship from alumni so students can learn from those working in other countries. Include topics like climate change and digital health, especially digital tools for budgeting and planning.

Section 2.5 – Broader Impact and Reflection

Has your view of the role of surgery in public health changed?

The program has absolutely influenced how I see surgery in public health. I now prioritize advocating for integrated systems to address surgical access, equity, and prevention.

Would you recommend the course to colleagues?

I would highly recommend the course. It equipped me professionally, gave me tools to make a tangible impact, and showed me how theory and practice together can transform systems.

Any final reflections:

The course reignited my passion for global health. It gave me a broader perspective and shaped my thinking to always consider the bigger picture of how people contribute to system change. I’m grateful for the networks I’ve built and the continued support from colleagues.

**Participant P4 Interview Transcript**

Participant Demographics:

- Age group: 20–29

- Gender: Female

- Professional background: Academic and research, policy

- Country of origin: South Africa

- Current workplace setting: University / Urban hospital

- Years of experience: 0–5 years

- Year of Course Completion: 2023

Section 2.1 – Background and Motivation

Professional background and reason for enrolling in the course:

I did my undergrad in pure science, microbiology and biochemistry. I wanted to become a doctor but never got into medical school. I decided to do infectious disease research instead. I moved into epidemiology and went to UCT for my Master’s in Public Health. While there, I worked with a professor at a children's hospital, which made me feel closer to neurosurgery. I saw many cases of violence in children and realised the importance of access to care. The professor suggested I look into global surgery and introduced me to another professor. I joined the course in my second year, and that’s when my career trajectory changed.

Expectations before joining the course:

At first, I didn’t understand what global surgery was. I thought only surgeons could do it. After the third lesson, I realised multiple stakeholders are involved. I had almost no expectations initially; I joined based on a professor’s recommendation. Once I understood, I wanted to learn how to use global surgery to inform policy and work with different people to make a difference.

How you first heard about the course and what influenced your decision to join:

I heard about it from a professor. I had to get permission to take it because I was in the epi and biostats track, so it didn’t count toward my degree. I took global surgery essentially as an extra course.

Section 2.2 – Learning Experience and Curriculum

Most useful or memorable curriculum components:

I enjoyed having experts in specific fields teach different topics. For example, policy was taught by someone with policy expertise, and HR management by someone experienced in that area. It was interactive with many activities. The policy section was challenging but valuable—it was the first time I understood how the South African policy system worked.

Relevance to previous or current responsibilities:

It didn’t relate much to my previous work, but after taking the course, I shifted entirely to global surgery. I now work at a Centre for Global Surgery, and it’s what I do daily.

Challenging aspects and how you managed them:

The policy section was challenging, and the fact that the course didn’t count toward my degree was frustrating. I still had to pay for it despite it not being accredited for my track. Timing was also an issue.

Moments of shift in perspective:

I realised you can work on something for decades without immediate results, but small changes—like improving surgical checklists—can still have an impact. It highlighted the complexity of health systems and the many factors involved.

Section 2.3 – Application and Professional Impact

How you have applied the knowledge/skills gained:

I coordinated the Global Surgery Summer School course at another university, using what I learned from the UCT course to design the content. I also apply what I learned daily in my global surgery research.

Changes in your role since completing the course:

I now run two projects independently, though I’m still a research assistant. I’ve had more opportunities to connect with leaders in the field, which is helping me grow professionally.

Examples of current projects:

1. A multi-country study on equitable access to healthcare, focusing on interpersonal violence.

2. A systematic review on lower limb amputations in low- and middle-income countries.

3. Mapping delays to care for violence victims in a metro area.

Incorporating surgical systems knowledge in daily work or decisions:

I understand the need for an integrated, multi-sector approach. You can’t impose changes without buy-in from stakeholders at all levels, and solutions must consider existing system constraints before policy changes can be made.

Section 2.4 – Program Delivery and Suggestions

How you found the learning format and delivery:

Having experts teach their specific areas worked very well. The only improvement would have been to make it count toward my degree at the time, which I believe has now been addressed.

Any barriers or difficulties faced during the course:

Balancing full-time work and full-time study was very challenging. I’m not sure how I managed it, but it was exhausting.

Suggestions for strengthening the course:

Adding an excursion would be valuable—either to a policy meeting, a hospital walkthrough, or another real-world setting so students can see surgical systems in action.

Section 2.5 – Broader Impact and Reflection

Has your view of the role of surgery in public health changed?

Yes. I became aware of the large divide not only between countries but also within South Africa, especially rural vs urban healthcare. I also learned about the role of traditional healers and the importance of understanding, rather than dismissing, cultural practices in surgical care.

Would you recommend the course to colleagues?

Yes, definitely. It changed my career, and global surgery education is rare in South Africa. UCT’s MPH course is one of the few comprehensive options available.

Any final reflections:

No additional points—most of my thoughts have already been expressed.

**Participant P5 Interview Transcript**

Participant Demographics:

- Age group: 40–49

- Gender: Female

- Professional background: Anthropology, NGO lead and industry/private sector

- Country of origin: United Kingdom

- Current workplace setting: NGO and other (creative workshop/jewellery business)

- Years of experience: 16+ years

- Year of Course Completion: 2023

Section 2.1 – Background and Motivation

Professional background and reason for enrolling in the course:

I studied anthropology and economics; spent time volunteering in South Africa with NGOs and special care centers; involved in income-generation projects for people with disabilities and chronic illnesses; eventually joined the NGO board; chose MPH to continue study and visa requirements; interested in global surgery after hearing a talk from a professor; had personal exposure to surgery which motivated curiosity.

Expectations before joining the course:

Expected a standard MPH module; wanted to learn about including surgery in public health interventions.

How you first heard about the course and what influenced your decision to join:

Heard at induction; scheduling conflicts almost prevented participation, but course coordinator’s flexibility and support encouraged enrollment.

Section 2.2 – Learning Experience and Curriculum

Most useful or memorable curriculum components:

Implementation science was most valuable; also epidemiology, human rights, health policy, and systems theory; valued guest lecturers and collaborative exercises, especially designing interventions.

Relevance to previous or current professional responsibilities:

Learned to approach surgical issues systemically; discovered gaps in referral processes for children with disabilities; applying learnings to improve policy and care at the NGO.

Challenging aspects and how you managed them:

Not particularly challenging; expectations were clear; workload manageable; assignments varied and skill-building.

Moments of shift in perspective:

Confirmed that public health tends to focus on “quick interventions,” but global surgery highlights the importance of surgical interventions for morbidity and mortality; learned how to effect change in complex systems.

Section 2.3 – Application and Professional Impact

How you have applied the knowledge/skills gained:

The course gave me a lot more confidence, even though I am not a surgeon. It helped me start asking questions in my NGO work and insist on improvements. While it didn’t directly apply to surgery, it helped me evaluate systems and processes critically.

Changes in your role since completing the course:

Not officially, but I’ve become more effective in my role. I am more involved and less willing to accept the first answer I’m given.

Incorporating surgical systems knowledge in daily work or decisions:

The course improved my understanding of complex systems. I now apply systems thinking to anticipate problems, create processes to prevent mistakes, and ensure consistent communication. This approach is used both in leadership at my NGO and in business operations.

Section 2.4 – Program Delivery and Suggestions

How you found the learning format and delivery:

Strengths: Sticking to timetables, continuity and context in guest lectures, a variety of learning formats (presentations, intervention plans, take-home exams), practical rather than memorization-focused assignments.

Areas for improvement: Course outlines and readings should be provided earlier (more than two weeks before start), to help with planning, especially for students balancing work and study.

Any barriers or difficulties faced during the course:

Balancing work and study is always hard, but deadlines were clear with no surprises, which made it manageable.

Suggestions for strengthening the course:

Linking students with real-life examples of what they are studying is highly beneficial (e.g., attending clinics). Formalizing this exposure would enhance the learning experience.

Section 2.5 – Broader Impact and Reflection

Has your view of the role of surgery in public health changed?

Absolutely. Surgery is often underrepresented in public health education. The course provided a broader pan-African perspective on surgical needs, emergency surgeries, and systems. It highlighted what is being missed and what can be done to improve provision.

Would you recommend the course to colleagues?

Yes, absolutely. The course teaches systems thinking and implementation science, which are broadly applicable beyond surgery. It is highly valuable and could even be considered mandatory for most streams.

Any final reflections:

The course provided confidence at a critical point in my NGO career. It helped me realize I can enact meaningful changes, manage systems more effectively, and achieve small victories that cumulatively make a difference.

**Participant P6 Interview Transcript**

Participant Demographics:

- Age group: 20–29

- Gender: Female

- Professional background: Clinical practice (Medical Student)

- Country of origin: South Africa

- Current workplace setting: University, urban hospital, and rural hospital

- Years of experience: 0–5 years

- Year of Course Completion: 2025

Section 2.1 – Background and Motivation

Professional background and reason for enrolling in the course:

I’m a fourth-year MBChB student. I got involved in a surgical society's global surgery and research portfolio, where I learned about global surgery and got involved in research, talks, and workshops. I later became head of that portfolio and worked with a surgery division on events. I also joined a Research Advocacy and Innovation Fellowship. During a fellowship meeting, a professor invited us to apply to the MPH global surgery track, which led me to apply. Now I’m in my first year of the MPH.

Expectations before joining the course:

I expected to gain a deeper understanding of global surgery, especially policy and frameworks. I came in with enthusiasm but little concrete knowledge, so I wanted to learn how global surgery actually translates into practice.

How you first heard about the course and what influenced your decision to join:

I first heard about it during a research fellows meeting. I didn’t know you could do it concurrently with MBChB. The main factor was my involvement in the Division of Global Surgery; it felt like the natural next step.

Section 2.2 – Learning Experience and Curriculum

Most useful or memorable curriculum components:

Not one specific component, but the format of teaching. I enjoyed the small group discussions with classmates from diverse backgrounds, applying readings to discussions, and developing critical thinking. It was different from undergrad lectures and exams—more interactive and enriching.

Relevance to previous or current responsibilities:

I can’t fully apply the tools yet as a student, but I use the course’s perspective when approaching patients. I think beyond diagnosis and treatment—considering health systems, access, and broader contexts. It changes the questions I ask and how I think about management.

Challenging aspects and how you managed them:

Everything was new, from lectures to readings to assignments. The shift from undergrad learning to research writing was challenging but valuable. Time management was also tough at first, balancing hospital work with evening lectures, but I adapted.

Moments of shift in perspective:

It wasn’t one moment but a cumulative shift. Coming in, I had a narrow view of surgery in public health. The course kept adding layers of knowledge and showed me how interconnected surgery is with health systems and public health.

Section 2.3 – Application and Professional Impact

How you have applied the knowledge/skills gained:

I’ve become an advocate for global surgery—sharing perspectives with peers, professors, and doctors, and contributing new angles in discussions and group projects.

Changes in your role since completing the course:

Not yet. I’ve only completed one half-semester course, so no major changes so far.

Incorporating surgical systems knowledge in daily work or decisions:

By considering broader contexts when seeing patients, and using what I’ve learned to explore topics further through academic reading and reflection.

Section 2.4 – Program Delivery and Suggestions

How you found the learning format and delivery:

I appreciated the interactive format, with discussions and prompts that encouraged collaboration. The open communication with conveners was great. We also had opportunities outside class, like attending surgical debates. Evening lectures worked well for part-time students. However, this semester’s daytime lectures are harder to manage for those working or studying full-time.

Any barriers or difficulties faced during the course:

Access to resources was fine. The main challenge was balancing time, especially with medical studies.

Suggestions for strengthening the course:

More practical, in-class applications of concepts (mini-assignments, scenarios, discussions). More content on stakeholders in global surgery and how to engage with them in real-world projects.

Section 2.5 – Broader Impact and Reflection

Has your view of the role of surgery in public health changed?

Yes. I used to see surgery as a siloed, last-resort intervention. Now I see it as integral to public health, linked to primary healthcare, economics, and all parts of the health system.

Would you recommend the course to colleagues?

Definitely. I think it should be compulsory for all medical and surgical trainees. Global surgery knowledge is essential for functioning as a doctor in South Africa’s complex health system.

Any final reflections:

The course has solidified my commitment to global surgery. It showed me this can be a concrete, long-term career path, not just an interest. It gave me skills and knowledge to apply practically and be hands-on in the space.

**Participant P7 Interview Transcript**

Participant Demographics:

- Age group: 40–49

- Gender: Male

- Professional background: Non-governmental organization (Engineering)

- Country of origin: South Africa

- Current workplace setting: NGO

- Years of experience: 11–15 years

- Year of Course Completion: 2023

Section 2.1 – Background and Motivation

Professional background and reason for enrolling in the course:

I was a consulting engineer for 15 years before switching to a non-profit in 2015, managing healthcare infrastructure projects in LMICs. I enrolled to better understand how to integrate engineering with global surgery, especially after the Lancet Commission report highlighted the link between infrastructure and surgical access.

Expectations before joining the course:

I had no major expectations as I was already experienced in the field. I wanted to learn academic practices for linking my project management skills to global surgery and enjoyed working with the diverse, multidisciplinary class.

How you first heard about the course and what influenced your decision to join:

I met a professor at a prior 3-day UCT course and stayed in touch. When I heard about this course, I took it as part of my Master of Public Health to reinforce my skills.

Section 2.2 – Learning Experience and Curriculum

Most useful or memorable curriculum components:

1) Working in multi-stakeholder teams with clinicians, which is directly applicable to my work. 2) The final project of designing and presenting a surgical intervention proposal.

Relevance to previous or current responsibilities:

It helped me understand clinicians' challenges, which improves how I run workshops, design projects, and interpret their needs for infrastructure work.

Challenging aspects and how you managed them:

The time commitment while working full-time was challenging. Learning to contribute to a group without always leading was a new and good experience.

Moments of shift in perspective:

Yes. I learned that small, specific interventions can have a significant long-term impact. You don't always need a big, flashy project to improve the surgical system.

Section 2.3 – Application and Professional Impact

How you have applied the knowledge/skills gained:

I'm more tuned into the challenges faced by clinicians and administrators. It has developed my empathy and helps me connect on a more patient-centric level in multi-stakeholder settings.

Changes in your role since completing the course:

Not directly. I changed roles just before the course. It hasn’t changed what I do, but it has definitely affected how I do my job, making me better at it.

Incorporating surgical systems knowledge in daily work or decisions:

I use it to better interpret clinicians' challenges and translate them into infrastructure solutions. I also now consider the broader regional or national impact of a project on the surgical ecosystem.

Section 2.4 – Program Delivery and Suggestions

How you found the learning format and delivery:

The small, intimate class size and access to diverse, senior faculty worked very well. The sharp learning curve for someone without base knowledge could be a challenge.

Any barriers or difficulties faced during the course:

The work-study balance was the main difficulty, especially when traveling for work meant missing lectures.

Suggestions for strengthening the course:

It was incredibly well-rounded. Someone from a clinical background might want more intervention-specific planning, but from my perspective, it was excellent.

Section 2.5 – Broader Impact and Reflection

Has your view of the role of surgery in public health changed?

Yes, it reinforced that fixing surgical delivery fixes many other parts of the health system. Surgery is a fundamental component that is often overlooked.

Would you recommend the course to colleagues?

Absolutely, especially for anyone in healthcare design touching on surgery. It’s important to understand the systemic issues, and this course combines policy, programmatic, and clinical aspects perfectly.

Any final reflections:

I’m very happy I took it. UCT has been progressive in integrating global surgery into public health. The faculty believes in what they are doing, and the well-researched methodologies were beneficial across the board.

**Participant P8 Interview Transcript**

Participant Demographics:

Age group: 30–39

Gender: Female

Professional background: Clinical practice (Speech Therapist and Audiologist)

Country of origin: South Africa

Current workplace setting: Non-governmental organization (NGO)

Years of experience: 6–10 years

Year of Course Completion: 2025

Section 2.1 – Background and Motivation

Professional background and reason for enrolling in the course:

As a speech therapist and audiologist working in both private and public healthcare facilities in South Africa, I frequently support patients requiring surgical interventions, such as grommet insertions or tonsillectomies. Observing wait times often exceeding 18 months for these procedures highlighted significant barriers to timely surgical care. This experience inspired me to enroll to deepen my understanding of surgical healthcare disparities and advocate for policy changes to improve access to equitable surgical care for my patients and underserved communities.

Expectations before joining the course:

Upon enrolling, I anticipated gaining a comprehensive understanding of global surgery as an interdisciplinary field. I sought to explore its core principles, the policies shaping surgical care access, and my potential role as an allied healthcare professional in addressing these challenges. The course successfully met these expectations, providing clear and engaging insights into surgical systems strengthening and health equity.

How you first heard about the course and what influenced your decision to join:

I first learned about the MPH Global Surgery track through a conversation with a colleague while working in an underserved community. This discussion prompted me to research UCT's MPH tracks, and I was particularly drawn to the Global Surgery track due to its innovative focus on improving surgical care access in resource-limited settings.

Section 2.2 – Learning Experience and Curriculum

Most useful or memorable curriculum components:

The lecture on citizen science stood out as particularly impactful, as it introduced innovative approaches to community-driven healthcare solutions. Additionally, the interactive group and individual activities incorporated into each session were highly engaging, fostering critical thinking and practical problem-solving skills.

Relevance to previous or current responsibilities:

The content provided valuable insights into the systemic reasons behind prolonged surgical waiting lists, a challenge I frequently encounter in my work. The course illuminated the global disparities in surgical care access, reinforcing my belief that surgery is a fundamental human right that should be equitably available to all.

Challenging aspects and how you managed them:

I found the course to be highly enriching and well-structured, with no significant challenges hindering my participation. The supportive learning environment allowed me to fully engage with the material.

Moments of shift in perspective:

The cumulative impact of the course profoundly shifted my perspective. The course highlighted the shared challenges in accessing surgical care, not only in South Africa but globally, particularly for underserved populations. Learning about the systemic barriers and inequities reframed my understanding of my role as an allied healthcare professional, inspiring me to consider how I can contribute to advocacy and policy changes.

Section 2.3 – Application and Professional Impact

How you have applied the knowledge/skills gained:

As a full-time student, I have not yet had the opportunity to directly apply the knowledge and skills gained in a professional setting. However, the insights I've acquired are shaping my academic pursuits and preparing me to contribute meaningfully to surgical care improvements in the future.

Changes in your role since completing the course:

As I am currently a full-time student, my professional role has not changed since completing the course. I am focused on continuing my studies within the MPH program, with the goal of leveraging the knowledge gained to influence my future career.

Incorporating surgical systems knowledge in daily work or decisions:

As a full-time student, I am not currently engaged in daily professional work or strategic decision-making. However, the knowledge gained is informing my academic perspective and future career aspirations, particularly in understanding how to address surgical system challenges through collaborative and equitable approaches.

Section 2.4 – Program Delivery and Suggestions

How you found the learning format and delivery:

The learning format and delivery were engaging and effective, with diverse guest lecturers providing a broad range of perspectives that enriched the learning experience. The inclusion of interactive group and individual activities in each session was particularly impactful.

Any barriers or difficulties faced during the course:

I did not encounter any significant barriers or difficulties while completing the course. The program’s structure and support systems were conducive to learning.

Suggestions for strengthening the course:

To further enhance the impact, incorporating perspectives from a broader range of healthcare professionals, such as nurses and allied healthcare workers like occupational therapists or pharmacists, would be valuable. Providing clearer guidelines on assignment expectations and raising the complexity of some tasks to better align with master's-level expectations could also strengthen the learning experience.

Section 2.5 – Broader Impact and Reflection

Has your view of the role of surgery in public health changed?

The course has profoundly shaped my perspective on the critical role of surgery within public health and health system strengthening. I gained a deeper understanding of the systemic barriers that limit access to surgical care. The program highlighted surgery as an essential component of equitable healthcare, emphasizing its integration into public health frameworks to address disparities.

Would you recommend the course to colleagues?

While the course offers valuable insights, I would cautiously recommend it to colleagues in allied healthcare fields like speech therapy and audiology, as the program is still developing as a pioneering track. The broad scope provided a solid foundation, but as a professional seeking clear career pathways, I found it challenging to discern specific opportunities this course might unlock post-graduation. For colleagues interested in exploring interdisciplinary approaches to healthcare equity, this course could be enriching, but clearer guidance on its applicability to non-surgical fields would enhance its relevance.

Any final reflections:

As a full-time student who worked diligently to fund my participation in this first-of-its-kind program, I am both excited and uncertain about the opportunities it may create. The course has broadened my understanding of global surgical challenges and deepened my commitment to healthcare equity. However, as the program is in its early stages, the specific career pathways it offers remain unclear. I remain optimistic that the knowledge and perspectives gained will open doors to contribute meaningfully to surgical systems strengthening.

**Participant P9 Interview Transcript**

Participant Demographics:

Age group: 40–49

Gender: Male

Professional background: Clinical practice (Surgeon), Academic/Research, Public Health & Policy

Country of origin: Malawi

Current workplace setting: University / Academic Hospital

Years of experience: 16+ years

Year of Course Completion: 2022

Section 2.1 – Background and Motivation

Professional background and reason for enrolling in the course:

I trained as an obstetrician and gynaecologist, later subspecialising in urogynaecology. My work integrates clinical excellence with system-level leadership. As Head of a surgical unit at an academic hospital, I face the daily reality that even the best-trained surgeons cannot achieve equity if systems are not ready: limited theatre access, geographic barriers, delayed referrals, and under-resourced training pipelines. Enrolling in the MPH Global Surgery course allowed me to build health systems competencies, sharpen my implementation and advocacy skills, and anchor my PhD research in frameworks that inform sustainable, scalable change.

Expectations before joining the course:

My expectations were to gain a structured framework to analyse and strengthen surgical systems, understand how to translate research into policy and practice, develop leadership and advocacy skills to manage change across academic, clinical, and policy spheres, and build connections with peers and mentors across Africa working in global surgery.

How you first heard about the course and what influenced your decision to join:

I learned about the program through UCT’s global surgery community, especially colleagues. What convinced me was its applied orientation: bridging theory with practice, rooted in LMIC realities. It was not just about learning, but about shaping tools I could immediately apply to my unit leadership, outreach programs, and PhD research.

Section 2.2 – Learning Experience and Curriculum

Most useful or memorable curriculum components:

The modules on Health Systems Strengthening, Innovation, Implementation, Resource Management, and Leadership were all critical. Specifically, Health Systems Strengthening gave me the ability to think beyond individual procedures to system readiness. Asset mapping helped me frame advanced surgery not just as high-tech but as a scalable workforce strategy. Leadership and change management were transformative—helping me negotiate theatre access, engage ministries, and lead cross-disciplinary teams.

Relevance to previous or current responsibilities:

Every component mapped onto my roles. As Head of Unit, I applied systems thinking to improve perioperative care and staff workflows. As a national proctor, I used project management and change frameworks to design structured training. As a researcher, I used equity and systems frameworks to embed geospatial access analyses into policy conversations.

Challenging aspects and how you managed them:

The main challenge was balancing the course with a demanding portfolio: high-volume surgery, teaching, PhD revisions, and leadership responsibilities. I addressed this by integrating coursework into live problems (e.g., course assignments became dashboards or service redesign plans for my unit), applying leadership skills from a parallel executive course, and strict time blocking and delegation.

Moments of shift in perspective:

Key moments included realizing that small, data-driven changes (checklists, enhanced recovery pathways, dashboards) can produce dramatic system improvements. I began seeing surgery not as an isolated intervention, but as an essential public health service requiring systems design and monitoring. I also recognized that equity requires geospatial planning—without data-driven outreach, women in rural areas remain invisible.

Section 2.3 – Application and Professional Impact

How you have applied the knowledge/skills gained:

I have applied the knowledge extensively: I designed a competency-based MPhil in my surgical subspecialty, aligning training with service outcomes. I proctored a national robotic surgery training program, ensuring structured progression and capacity building. I introduced clinical dashboards tracking key performance indicators and used them in audit meetings for feedback loops. I also used geospatial access analysis to plan equitable outreach roadshows.

Changes in your role since completing the course:

My leadership as Head of Unit has been consolidated. I have become a national proctor for a advanced surgical platform. I have increased contributions to international guidelines and publications, and taken on greater supervision of fellows and trainees, building the next generation of surgeons.

Incorporating surgical systems knowledge in daily work or decisions:

I use key performance indicator dashboards for decision-making regarding theatre allocations and complication monitoring. I apply systems thinking in aligning training with service outcomes. I use advocacy skills to engage ministries, hospital managers, and funders. I apply change management to introduce new protocols, training, and data systems.

Section 2.4 – Program Delivery and Suggestions

How you found the learning format and delivery:

The modular structure, strong mentorship, and real-world assignments worked very well.

Any barriers or difficulties faced during the course:

The heavy clinical and academic workload was a significant barrier, as were data access challenges in LMIC contexts. I overcame these with asynchronous learning, structured delegation, and using practice datasets.

Suggestions for strengthening the course:

I would suggest adding implementation labs with live datasets and ready-to-use toolkits (e.g., for costing, business cases, stakeholder templates). Additional topics could include financing & commissioning for surgical services, digital health and AI in global surgery, workforce planning and task-sharing models, and expanded global diplomacy & advocacy modules with case-based simulations.

Section 2.5 – Broader Impact and Reflection

Has your view of the role of surgery in public health changed?

The course cemented surgery as a public health priority, requiring financing, governance, monitoring, and equity frameworks. It also reinforced that maternal health and complex surgical conditions depend not only on skilled clinicians but on system readiness, including intrapartum care, referral pathways, and surgical access.

Would you recommend the course to colleagues?

Yes, unequivocally. It is one of the few programs that delivers practical, system-level skills that clinicians and academics can immediately apply. It bridges the gap between individual training and scalable models of service delivery.

Any final reflections:

The course, alongside my PhD in Global Surgery and executive leadership training, created a coherent framework for impact based on Evidence (research), Design (frameworks), and Execution (leadership). This triad now underpins my work: developing scalable surgical models that integrate training, equity, and innovation to transform women’s health care in South Africa and Malawi.

**Participant P10 Interview Transcript**

Participant Demographics:

Age group: 20–29

Gender: Female

Professional background: Clinical practice (Medical Doctor)

Country of origin: South Africa

Current workplace setting: Urban Academic Hospital (Trauma Surgery Unit)

Years of experience: 0–5 years

Year of Course Completion: 2025

Section 2.1 – Background and Motivation

Professional background and reason for enrolling in the course:

I am a medical doctor working in the Trauma Surgery Unit at a large academic hospital. My community service was in a rural emergency department. I have witnessed first-hand the systemic challenges in accessing and delivering timely surgical care, from resource shortages to long waiting lists. These experiences highlighted the critical need for system-level solutions. I have always had a passion for contributing to health systems beyond the front room from a systems-level approach. This motivated me to pursue the MPH in Global Surgery to develop the skills to drive surgical systems innovation and contribute to equitable surgical care for underserved populations.

Expectations before joining the course:

I expected the course to broaden my understanding of the global and systemic dimensions of surgical care, beyond the bedside. Specifically, I wanted to learn about surgical systems strengthening, policy frameworks, and innovative approaches to bridging inequities in access to safe surgery. These expectations were met, as the course provided a deeper understanding of how my clinical role connects to larger global health priorities.

How you first heard about the course and what influenced your decision to join:

I first learned about the MPH Global Surgery program through the MPH website, as I had initially planned on doing an MPH in health systems, but this track was better suited to my specific interests. The uniqueness of the program, with its strong focus on health equity and surgical systems in LMICs, aligned closely with my experiences in trauma care. My decision to enroll was shaped by a desire to merge clinical experience with health systems knowledge.

Section 2.2 – Learning Experience and Curriculum

Most useful or memorable curriculum components:

The components on health systems analysis and citizen-science implementation were particularly impactful. The surgical ecosystem framework assisted with conceptualizing the vastness and complexity of Global Surgery. The curriculum provided frameworks for thinking beyond individual patient care and toward scalable interventions. The group work and interactive sessions also encouraged collaboration across disciplines.

Relevance to previous or current responsibilities:

The course directly relates to my daily work in Trauma Surgery. I often see patients whose outcomes are shaped not only by surgical skill but by systemic barriers such as delayed access, lack of capacity, and inequitable distribution of resources. The course gave me the language, frameworks, and evidence base to contextualize these multiple challenges. I now approach these challenges by considering long-term sustainable solutions.

Challenging aspects and how you managed them:

The biggest challenge was balancing the clinical demands of working in trauma surgery with academic coursework. However, the relevance of the content to my daily practice motivated me to stay engaged, and the interactive learning environment made it easier to connect theory to practice. Overall, the course itself is well-structured with a lot of support.

Moments of shift in perspective:

A pivotal moment was realizing that surgery is not only a clinical intervention but a fundamental component of public health, affecting millions of patients. Understanding that surgical conditions account for a significant portion of the global disease burden and its long-term economic impact shifted my perspective on how essential it is to integrate surgery into national and global health priorities. Realising that the largest part of helping surgical patients goes beyond the operating room is a perspective I now apply to most of my work challenges.

Section 2.3 – Application and Professional Impact

How you have applied the knowledge/skills gained:

I have started applying systems-thinking approaches in trauma care—for example, identifying process gaps in patient flow and transfer and discussing possible innovations with colleagues. I also approach clinical challenges with a broader view, considering how health policy, standard operating procedures and system design influence the care I deliver at the bedside.

Changes in your role since completing the course:

My role as a Medical Officer remains clinically focused, but I have increasingly taken on informal leadership in quality improvement discussions within the trauma unit. I am also beginning to align myself with research projects in global surgery and surgical systems improvement through my MPH program.

Incorporating surgical systems knowledge in daily work or decisions:

I incorporate systems awareness into my decision-making by considering not just the immediate surgical intervention but also how patient outcomes are shaped by broader structural issues—such as referral pathways, access to theatres, and resource allocation. This helps me advocate for changes that can improve patient flow and outcomes through improved management.

Section 2.4 – Program Delivery and Suggestions

How you found the learning format and delivery:

The format was engaging, with diverse lecturers and classmates, and interactive sessions that brought theory to life. The cross-disciplinary approach enriched discussions. However, more case studies from LMIC trauma and surgical contexts would make the content even more relatable to frontline clinicians like myself.

Any barriers or difficulties faced during the course:

Balancing the demands of clinical work in trauma surgery with postgraduate studies is challenging. Nonetheless, the program’s supportive structure and relevance to my daily work helped me navigate these challenges effectively.

Suggestions for strengthening the course:

Including more content on surgical innovation and implementation science in LMIC settings would strengthen the course. Practical tools for designing and evaluating system-level interventions would also be valuable for clinicians seeking to drive change – I am aware this would prolong the course, but it would benefit effecting real-time change.

Section 2.5 – Broader Impact and Reflection

Has your view of the role of surgery in public health changed?

Absolutely. The program reframed surgery in my mind from a specialty service to a cornerstone of universal health coverage and public health. I now see my role as not just providing trauma care, but as part of a broader movement to ensure surgery is accessible, safe, and equitable across all populations. I am more aware of my own skills that I need to develop to effect innovation and greater impact.

Would you recommend the course to colleagues?

Yes, I would strongly recommend it to colleagues in surgery and trauma care. The course bridges the gap between clinical practice and health systems thinking, equipping clinicians with the tools to address systemic challenges and innovate within their contexts. We cannot address the high surgical patient volumes by only addressing the clinical aspect. I think this course should be essential for doctors to fully conceptualise the broad complexity of the work we do.

Any final reflections:

The course has been transformative. It has given me a framework to connect my passion for trauma surgery with a vision for health system improvement and innovation. It has inspired me to think of my career not only in terms of patient care but also in terms of contributing to structural change in surgical care delivery in South Africa and across LMICs.
