# Supplementary material for: A qualitative evaluation of a global surgery course within the University of Cape Town’s master of public health curriculum: A cross-sectional study
Source: PLOS Glob Public Health. 2025 Dec 12;5(12):e0005646. doi: 10.1371/journal.pgph.0005646 (PMC12700414; doi:10.1371/journal.pgph.0005646)
Supplement: Attachment — Submitted filename: 20251015-Review Comments.docx [file pgph.0005646.s004.docx]

**Overall comment:**

The manuscript is clearly written, logically structured, and well-referenced. The topic of integrating global surgery into an MPH curriculum is timely and relevant, particularly within African public health training contexts. However, the paper significantly overstates the distinctiveness and real-world impact of this course. The described learning outcomes of peer learning, expert engagement, and professional development are not unique to this course. They align with the established design and objectives of the broader UCT MPH curriculum.

The claims of transformation and practical impact remain largely theoretical, with limited evidence of measurable outcomes or differentiation from other MPH specialisations. The course, while academically valuable, appears to deliver (in this early stage) a conceptual and knowledge-based enrichment rather than a transformative or practice-shifting experience. This is commendable.

However, I would suggest the authors provide a clearer comparative framing: how does this course differ from other UCT MPH courses / specialisations or from comparable global surgery or health systems programmes internationally? More importantly, how does its impact manifest relative to other specialisations i.e., what tangible professional or applied benefits would a student gain from selecting the Global Surgery track over others? Addressing these points would strengthen the paper’s credibility and help situate the course within the broader ecosystem of public health education, without overstating its influence.

**General**

1. The title reads as overstated and leans toward self-promotion. The term *“novel”* should be reconsidered or qualified. Including the institution’s name would also situate the work more appropriately and clarify context.
2. Global surgery is somewhat of a misnomer
3. Its hard to divorce away from the original nomenclature but its not accurate to speak of global surgery when in fact speaking about health systems / public health infrastructure / health economics… at best, surgery forms part… a critical and undermined part of the public health system
4. The manuscript substantially overstates the real-world impact of the course. In the South African context, the practical contribution of a global surgery course to strengthening health systems or addressing inequities in access to care remains limited and largely theoretical. While the course may expand conceptual and academic knowledge, the claim that it meaningfully transforms health system outcomes or national policy readiness is not supported. If there is evidence, it would be great to include it to the framing. However, its unlikely that that could be attributed to this specific course.

A more balanced framing would acknowledge that this course complements, rather than substitutes for, the work of public health professionals engaged in implementation, program design, and system reform.

**Specific**

1. **Line 108: Sub-Theme 2A: learning *from* and *with* peers**

This finding is not unique to this course. Peer learning is a central pedagogical feature of the broader MPH programme itself. If anything, this sub-theme may reflect the strength of the MPH structure, rather than a distinctive feature of the global surgery course.

It would be helpful for you to clarify whether the course provides any particular type of peer or stakeholder interaction that differs meaningfully from the rest of the curriculum.

2. **Line 115: Sub-Theme 2B: Learning From Experts**

Similar to the comment above, this is a standard feature of most high-quality MPH programmes globally. Learning from experts is not a unique or innovative element of this course, and the manuscript should be careful not to present it as such.

3. **Line 144: Theme 4: Professional impact: career confidence, catalysis, and creation**

This theme of professional impact reads as overstated and its not sufficiently benchmarked. These outcomes are common goals of any MPH programme. Without comparative analysis i.e., against other MPH tracks at UCT or against comparable global surgery programmes such as Harvard’s Program in Global Surgery and Social Change, the conclusions posited here about professional impact lack a robust basis.

Confounding factors need to be acknowledged as well. Most of the sample size appears to have medical or academic backgrounds that predispose them to relevant professional advancement, independent of this specific course.

4. **Line 201: Table 2 Reported Professional Application of Course Learning**

The professional outcomes described could plausibly result from the broader MPH curriculum rather than this single course. Because the course is integrated within the degree structure, it is methodologically difficult to attribute impact solely to this course without an appropriate comparator.

A qualitative study of the stand-alone version of this course (which used to exist), prior to integration into the MPH, would provide a clearer and more defensible assessment of its distinct contribution.

5. **Line 240-243: “The foundational novelty if this course…diverse professional backgrounds.”**

The claim that diversity is a defining novelty is not supported by the sample of participants. Based on the data presented, none of the respondents appear to represent the broad non-medical or cross-sectoral backgrounds implied. The sample (predominantly clinicians, researchers, and allied health professionals) limits the ability to generalize or claim inclusivity as a defining strength. The manuscript needs to clarify whether course entry is selective and, if so, how diversity is operationalized.

As written, it is difficult to reconcile the intended goals of diversity and interdisciplinarity with the evidence provided.

6. **Line 292-298: “Based on the findings of this study…and adapt the framework across diverse contexts.”**

This conclusion is an overreach. Much of the global surgery framework draws heavily from established public health principles e.g., systems thinking, health equity, policy analysis, and resource allocation. What distinguishes it [global surgery] is the surgical framing of these established ideas, not a new paradigm. The manuscript risks positioning global surgery as both the hub and the spoke of public health discourse, when its contribution is better understood as contextual adaptation of
