# Supplementary material for: A qualitative evaluation of a global surgery course within the University of Cape Town’s master of public health curriculum: A cross-sectional study
Source: PLOS Glob Public Health. 2025 Dec 12;5(12):e0005646. doi: 10.1371/journal.pgph.0005646 (PMC12700414; doi:10.1371/journal.pgph.0005646)
Supplement: Attachment — Submitted filename: Rebuttal Letter.docx [file pgph.0005646.s006.docx]

Rebuttal Letter

To: The Editors and Reviewers

From: The Authors

Date: 25^th^ October 2025

Manuscript ID: PGPH-D-25-02795

Dear Editors and Reviewers,

Thank you so much for your thoughtful and constructive feedback on our manuscript. We truly appreciate the time and care you took to review our work. Your comments were incredibly helpful and have guided us in making significant improvements to the paper.

We’ve worked hard to address every point you raised. Below, we have outlined how we responded to your specific suggestions.

Our Responses to Your Comments

**To Reviewer #2:**

Thank you for your encouraging words and for the very practical suggestions to improve the clarity and rigor of our manuscript.

On the title: You were right, the original title was a bit too narrow. We have changed it to better reflect that we studied a course within the larger MPH program. The new title is: "A Qualitative Evaluation of a Global Surgery Course within the University of Cape Town's Master of Public Health Curriculum: A Cross-Sectional Study."

On describing the program: This was a great point. We have added a new section in the Methods called "Program and Course Description" where we briefly outline the structure of the MPH, the details of the Global Surgery course, what it covers, and how it is taught.

On informed consent: Yes, we did obtain informed consent. We have now made sure to explicitly state that in the "Data Collection and Management" section, so it is clear to all readers.

On the interview guide: We have included the interview questions as an Appendix so readers can see the exact questions we asked.

On linking themes to surgery: This was a crucial insight. We have added brief introductory sentences to Themes 2 through 5 in the Results to explicitly tie them back to the context of learning global surgery.

**To Reviewer #3:**

Thank you for your positive feedback and for the helpful methodological notes.

On justifying our methods: We have added a sentence in the "Study Setting and Design" section to better explain why a qualitative approach was the best fit for our research question.

On recall bias: This is a very fair point. We have added a mention of recall bias as a potential limitation in the Discussion section.

**To the Reviewer who provided the overall and general comments:**

Thank you for your challenging and incredibly valuable feedback. You pushed us to think more critically about how we were positioning our work, and we agree that our initial claims were sometimes too strong. We have significantly revised the manuscript to be more balanced and precise.

On overstating distinctiveness and impact: This was our most important takeaway. We have gone through the entire manuscript and toned down the language, replacing words like "novel," "profound," and "transformative" with more accurate terms like "valuable," "notable," and "effective." We now frame the course as a successful application of public health principles to surgery, rather than a revolutionary new idea.

On peer learning and expert lecturers not being unique: You are absolutely correct. We have added text in the Discussion for Themes 2 and 4 to acknowledge that these are strengths of the broader MPH program, and that their value in our course was in how they helped students understand the multi-stakeholder reality of surgical systems.

On professional impact and attribution: This is a key limitation of studying a single course within a degree. We now explicitly state in the Discussion that while the course appears to have successfully delivered on core MPH objectives, we cannot attribute these outcomes solely to this one course and they likely result from the cumulative effect of the entire program.

On the concept of "Global Surgery": We have reframed this in the Introduction, defining it clearly as the application of public health principles to the surgical ecosystem, which we believe is a more humble and accurate description.

On the need for comparative framing: We have added a new paragraph in the Discussion that directly compares our course to other MPH specializations and other global surgery programs, positioning it as a complementary track within public health education.

On the diversity of the sample: You rightly pointed out that our sample, while multidisciplinary within health, was not as cross-sectoral as we might have implied. We have moderated our claims about this and added a sentence in the Limitations section acknowledging this point.

On the conclusions being an overreach: We have completely rewritten the Conclusions to be more measured, focusing on the course as a practical "blueprint" and a "valuable strategy" rather than a paradigm shift.

We have now explicitly stated in the 'Research Team, Reflexivity and Data Analysis' section that a completed COREQ checklist has been provided as an Appendix.

Thank you again for your guidance. We feel the process of revising this paper has been a fantastic learning experience for us, and we believe the manuscript is now much stronger, more credible, and more balanced. We are hopeful that you find it suitable for publication.

With sincere thanks,

On behalf of the Authors

Yvan Zolo
